# It is all about the insects: a retrospective on 20 years of forensic entomology highlights the importance of insects in legal investigations

**DOI:** 10.1007/s00414-021-02628-6

**Published:** 2021-09-30

**Authors:** Lena Lutz, Richard Zehner, Marcel A. Verhoff, Hansjürgen Bratzke, Jens Amendt

**Affiliations:** Institute of Legal Medicine, University Hospital Frankfurt, Goethe-University, Kennedyallee 104, 60596 Frankfurt am Main, Germany

**Keywords:** Postmortem interval, Legal medicine, Negligence, Myiasis, Blow flies

## Abstract

This study highlights the importance of insect evidence by evaluating 949 insect-associated cases, including 139 entomological reports, from 2001 to 2019 at the Institute of Legal Medicine Frankfurt/Germany. With a high number of cases in the summer months and a low number in the colder season, 78.5% of the bodies were found indoors, regardless of year or month. In more than 80% of the cases, where PMI information was available (n = 704), the presumed PMI ranged from 1 to 21 days, a period during which entomological evidence can provide a day-specific estimate of PMI_min_. In cases where insects have been identified to species level (n = 279), most bodies were infested by one or two species with a maximum of 10 different species. Overall, a total of 55 insect species were found. Information on biology, activity and distribution of the most abundant taxa is given and applied for 5 case histories estimating different PMI_min_s of up to over 6 months. Despite proved importance and scientific development of forensic entomology, insects are still rarely considered as a tool in forensic case work. The main reasons are a lack of awareness and (too) late involvement of a forensic entomologist. Our work shows that forensic entomology is an independent discipline that requires specialist expertise.

## Introduction

Forensic entomology analyses insect evidence to draw conclusions on legal matters [1]. Its value for forensic casework was only recognized at the beginning of the twentieth century [2], but from then on, decades of research have turned it into one of the most accurate and precise method used to establish the time since death in the later postmortem interval [2–7], especially when standard forensic medical approaches are no longer appropriate to determine the time since death [4]. Forensic entomology is mainly applied for estimating the minimum postmortem interval (PMI_min_), i.e. the time since the first insect colonization, by determining the age of insect stages developing on the human remains and analysing the successive patterns, pre-appearance [8, 9], arrival, residency [10, 11] and departure of insects from a carcass. However, it can be applied to many more areas and questions, e.g. by giving valuable hints for a cadaver relocation [12] or manipulation of a crime scene. Furthermore, larvae and pupae, more precisely their gut and tissue content, has promising information for the investigation of sexual crimes, such as rape [13, 14], especially when the victim was found in an advanced stage of decomposition [15], for genotyping human DNA to identify the source they fed on [16, 17] or for detecting drugs which were consumed by the deceased during life time [18, 19]. Last but not least, in times of climate change and further spreading of invasive species, the insect infestation of living people will be more common even in the Northern hemisphere and can be evaluated by analysing the fauna of the patient (to determine periods of neglect, to clarify questions of responsibility regarding neglect, etc.).

**Fig. 1:**
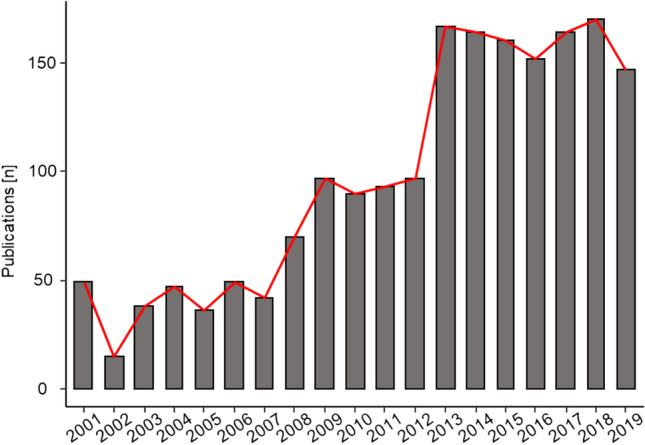
Number of publications with “forensic entomology” in the title, abstract or keywords published in peer-reviewed journals from 2001 to 2019

A search result in Google for “forensic entomology” yielded 2.190.000 hits (16.10.2020; 09:14), including research studies, newspaper articles, videos and interviews, proving the popularity of this research area. But despite the worldwide positive development and improvement in this field particularly in the last 20 years (Fig. 1), we note that insects are still too rarely considered as a tool in forensic case work. For Germany this is also reflected in the fact that at 41 national institutes of legal medicine only three employ forensic entomologists.

Obviously, there is an imbalance between the increasing scientific development and its usefulness and the actual consideration of forensic entomology in case work and its assignment by police and prosecution. In the present paper we highlight the importance of insect evidence in forensic casework by evaluating almost 1000 insect-associated cases (n = 949) from 2001 to 2019. Furthermore, we give an overview of the entomological reports (n = 139) during this period, and finally we discuss and question the possible obstacles that still prevent an adequate application.

## Material and methods

### Data basis

In order to obtain an overview of the number of insect-associated cases in Frankfurt, all reports of autopsies performed at the Institute of Legal Medicine Frankfurt am Main from the years 2001 to 2019 were evaluated. We searched for the keywords “insects”, “maggots”,” beetle”, “fly”,” pupae”,” puparia”, “infestation”. Using this method, we found 854 insect-associated cases. Also included in the analysis were 95 cases from all over Germany and other German speaking countries, namely Switzerland, Austria and Luxembourg, where an entomological report was prepared by at least one of the authors. In total, we included 949 insect-associated cases from the years 2001 to 2019 in the analysis. For each case, information was noted, if available, on the:

- Date (day, month, year) of the discovery and the autopsy
- Sex and age of the deceased
- Place of discovery (indoor or outdoor)
- Particularities of the place of discovery (trunk of a car, balcony, trashcan, flat, forest, open field, etc.)
- Presumed postmortem interval (PMI); based on the time of last seen alive or other indications such as newspapers, letters or statements from neighbours and relatives
- Manner of death (natural, unnatural, unclear)

The place of discovery was used to describe the accessibility of a body for insects, rather than just to describe the location. In this context a body found, e.g. on a balcony of an apartment, was classified as an outdoor case. The presumed PMI was classified in five groups (1–7 days, > 1–3 weeks, > 3 weeks–3 months, > 3–6 months, > 6 months).

### Entomological evidence

In almost 30% (n = 279) of the cases, entomological evidence, i.e. juvenile and adult stages of necrophagous insects, was sampled and identified. In most cases the sampling was performed only during the autopsy or at the scene of death. In some cases, both sampling at the scene of death and during the autopsy was possible. The sampling was performed, if possible, by a forensic entomologist, but sometimes a medical examiner or the police was responsible for the evidence collection. Diptera larvae were killed with almost boiling water and afterwards stored in 96% ethanol and/or transferred to minced meat and bred under controlled temperature in the laboratory until the adult stage. Adult beetles and flies as well as beetle larvae were killed by freezing at – 20 °C and afterwards stored in 96% ethanol. Species identification was performed on the basis of morphological characters with the current systematic literature [20–23] and voucher specimens from the Institute of Legal Medicine Frankfurt/Germany. Where no reliable identification based on morphological characteristics was possible, the mitochondrial *cytochrome c oxidase subunit I* (COI) gene was analysed. In most cases the universal PCR primers LCO1490 and HCO2198 [24] were used to amplify the commonly used 648-bp-long barcoding region. In some cases, only identification down to the genus level was possible, as only fragments of the species or, in cases for the flies, empty puparia were provided as evidence.

In ~ 15% of the cases, an entomological report was written. For these cases the PMI_min_ was calculated on the basis of the insect ID and reconstructed temperature data of the scene of death.

### Statistical analysis

A descriptive analysis of the number of insect-associated cases throughout the years 2001 to 2019 and for each month was performed. Furthermore, the percentage distribution of the presumed PMI classes and manner of death was analysed visually. Based on the entomological data a species list was build, the seasonal oviposition activity and the habitat preference (i.e. indoor, outdoor) of the most important species of forensic importance were graphically summarized. All data were analysed and charted with R version 3.6.2 [25].

## Results and discussion

### Case evaluation

**Fig. 2:**
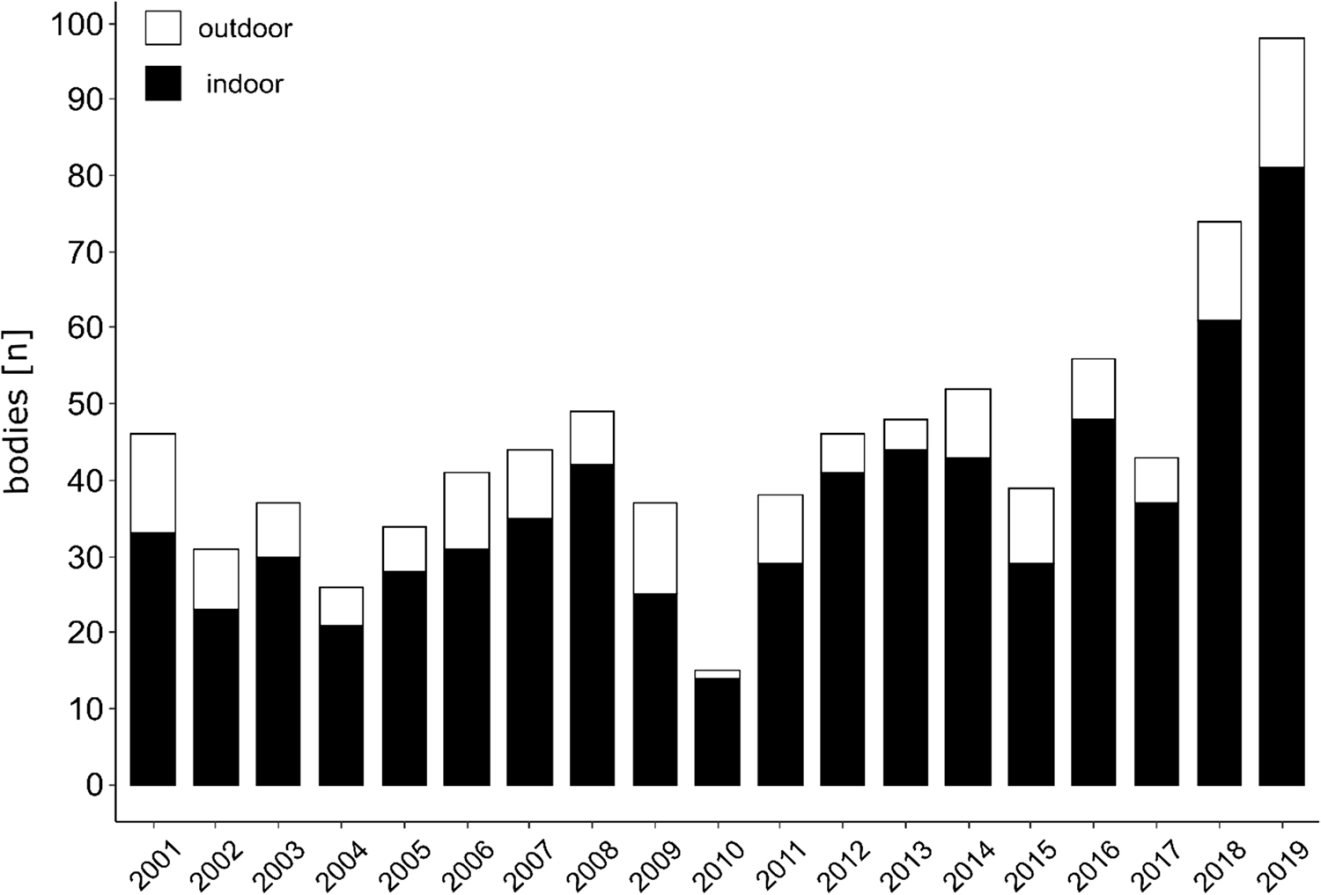
Number of bodies infested with insects (bars; n = 854) found indoor (black) and outdoor (white) in the period from 2001 to 2019

**Fig. 3:**
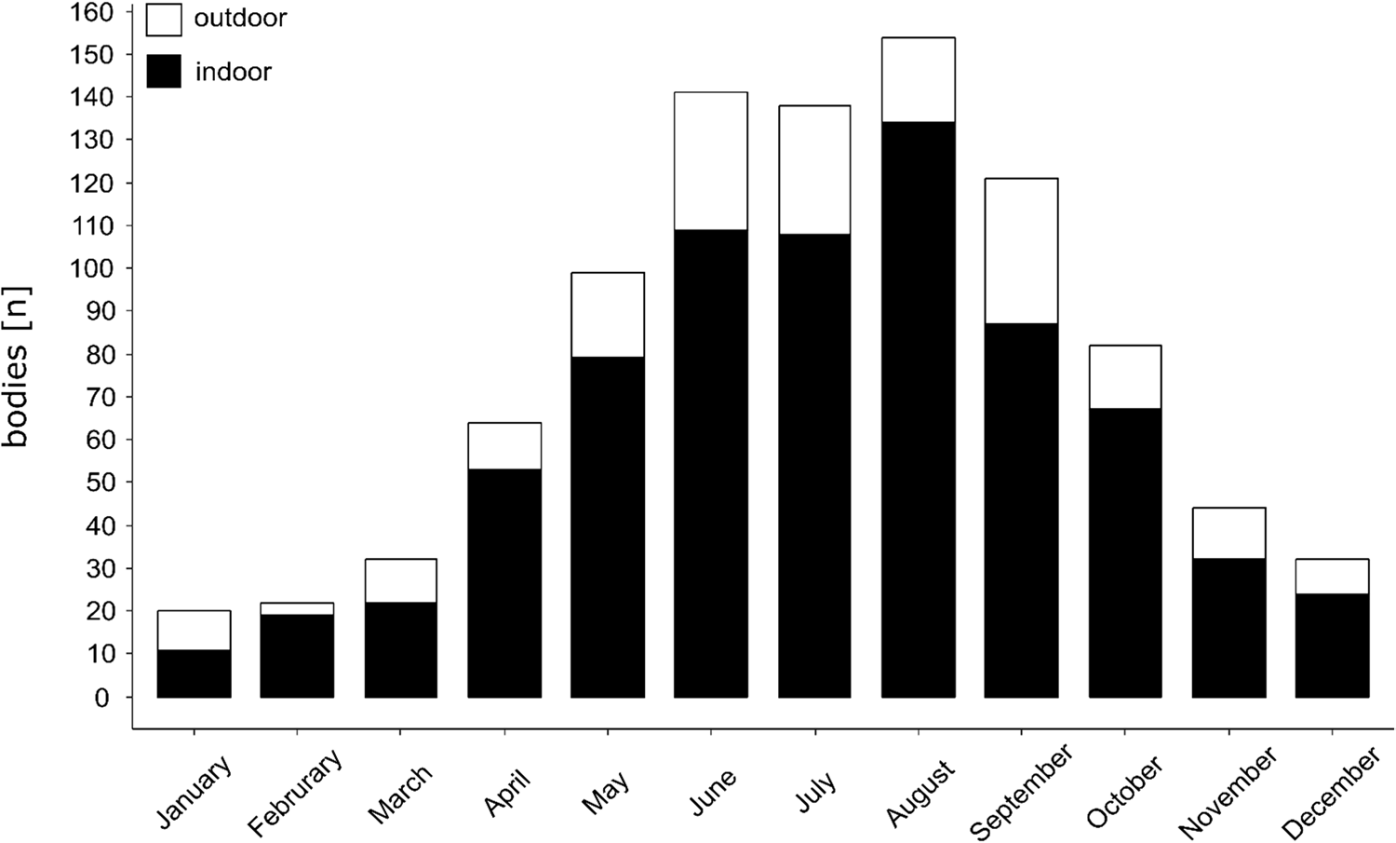
Number of bodies infested with insects (bars; n = 949) in the period of 2001 to 2019 found indoor (black) and outdoor (white) month-by-month

Overall, 949 insect-associated cases were evaluated (Table 1). The number of cases from the Institute of Legal Medicine Frankfurt (n = 854) varied between the years with a mean of 44 insect associated cases per year. However, the number increased from 2012 to 2019 with a maximum of 98 bodies in 2019 (Fig. 2). For all cases (n = 949) a seasonal trend could be observed, with a high number of cases in the summer months from May to September (Fig. 3) and a low number in the colder season from October to April. This trend was the same for all years (results not shown). In most cases, the bodies were found in an indoor scenario (78.5%, Table 1), regardless of year or month (Fig. 2, Fig. 3), while only a few (21.5%, Table 1) were found outdoors. The median age of the deceased was 56 years, and most of the bodies were male (69.9%, Table 1).

**Table 1:** Insect-associated cases from 2001 and 2019 with information of the place of discovery, the age and sex of the deceased; “information of insect species” indicates whether the insects were determined or whether only the insect infestation was documented

Bodies infested with insects	949
Information of insect species	29.5%
Entomological report	14.5%
Indoor	78.5%
Outdoor	21.5%
Male	69.9%
Female	30.1%
Age (mean)	55.6
Age (median)	56

In more than 80% (Table 2) of all cases where PMI information was available (n = 704), the presumed PMI ranged from 1 to 21 days (3 weeks), a period during which entomological evidence can provide a day-specific estimate of PMI_min_. In only a few cases, the bodies were found 4 to 6 (4.23%) or more than 6 months (4.09%) after death. In about one-third (35%, Table 2) of the cases, the person died of natural causes (e.g. heart failure, internal causes), and in 26% there was an unnatural type of death. This includes suicides (e.g. poisoning, drug abuse, gunshot wounds, stab wounds) as well as homicides. However, in most cases (38.71%, Table 2), no clear cause of death could be determined during the autopsy due to external and internal decay (autolysis) of the body.

**Table 2:** Percentage distribution of the presumed PMI and the manner of death for insect-associated cases from the years 2001 to 2019

PMI	704 (n)
1–7 d	31.73%
> 1–3 w	51.48%
> 3 w–3 m	7.76%
> 3–6 m	4.23%
> 6 m	4.09%
Manner of death	949 (n)
Natural	35.19%
Unnatural	26.10%
Unclear	38.71%

### Infestation rate and insect taxonomy

In cases where entomological evidence was sampled and insects identified (n = 279), most bodies were infested by one (30.11%) or two species (24.38%) with a maximum of 10 different species colonizing a carcass. Overall, mono-colonization occurred more often indoors than outdoors (results not shown). It cannot be ruled out that this pattern is/was biased by different factors such as the person who was responsible for the evidence collection (forensic entomologist, medical examiner, crime scene technician or police), the sampling location (scene of death, autopsy, scene of death and autopsy) or specific sample effort (e.g. sampling at a homicide site versus a natural cause of death in an apartment) and not only by the length of the PMI or preferences and predominance of single species. In a recent study [26], we highlight that all these factors have a major impact on the quality (e.g. species diversity or developmental stages found) of the entomological evidence, leading to a biased or limited view on the real species diversity on the cadaver.

A total of 55 insect taxa represented by three insect orders: Coleoptera (beetles), Diptera (flies) and Hymenoptera (sawflies, wasps, ants, bees) were found to colonize human remains. The highest species diversity, 13 families and 37 taxa, was observed in flies (Table 3) which was also the most important and abundant order. Overall, 16 beetle taxa in six families were found. For the Hymenoptera just two taxa ordered in two families were found. It must be critically questioned to what extent the beetle species reflect realistic numbers, especially since usually numerous species of Nitidulidae [12, 27] or Staphylinidae [28] are to be expected on a cadaver in the field. As an example, Weithmann et al. [29] determined 80 rove beetle species that were attracted to piglet cadavers across various forest stands in Germany. The possible “undersampling” of beetles in this study is partly due to the fact that the non-trained staff mainly look for fly maggots because there are more common and easier to spot and therefore may simply overlook the beetles, and partly due to the fact that the majority of cases were in an indoor scenario, where beetles are less common and abundant as outdoors. Since our aim was not an explicit analysis of species diversity on cadavers, we believe that a possible preference for Diptera has no negative impact on the assessment of insect evidence in the forensic sciences.

**Table 3:** Insect species (Coleoptera, Diptera, Hymenoptera) sampled from human bodies (n = 279) from 2001 to 2019. Information on the number of cases the species were found as well as the percentage distribution are given

Order	Family	Species	n	%
Coleoptera	Cleridae	*Necrobia ruficollis* (Fabricius, 1775)	3	1.08
		*Necrobia rufipes* (de geer, 1775)	3	1.08
	Dermestidae	*Dermestes lardarius* linnaeus, 1758	10	3.58
		*Dermestes haemorrhoidalis* küster, 1852	7	2.51
	Geotrupidae	*Anoplotrupes stercorosus* (scriba, 1791)	2	< 1
		Geotrupes sp.	1	< 1
	Histeridae	*Saprinus semistriatus* (scriba, 1790)	4	1.43
	Silphidae	*Necrodes littoralis* (Linnaeus, 1758)	8	2.86
		*Nicrophorus humator* (gled, 1767)	3	1.08
		*Nicrophorus vespilloides* (herbst, 1748)	3	1.08
		*Nicrophorus* sp.	4	1.43
		*Thanatophilus sinuatus* (fabricius, 1775)	2	< 1
		*Thanatophilus rugosus* (linnaeus, 1758)	1	< 1
		*Thanatophilus* sp.	3	1.08
	Staphylinidae	*Creophilus maxillosus* (linnaeus, 1758)	5	1.79
		*Aleochara curtula* (goeze, 1777)	1	< 1
		*Philonthus succicola* thomson, 1860	1	< 1
		*Philonthus politus* (linnaeus, 1758)	1	< 1
Diptera	Calliphoridae	*Lucilia sericata* (Meigen, 1826)	150	53.76
		*Calliphora vicina* robineau-desvoidy, 1830	111	39.78
		*Lucilia ampullacea* villeneuve, 1922	68	24.37
		*Protophormia terraenovae* (robineau-desvoidy, 1830)	55	19.71
		*Calliphora vomitoria* (linnaeus, 1758)	45	16.13
		*Phormia regina* (meigen, 1826)	45	16.13
		*Lucilia caesar* (linnaeus, 1758)	27	9.68
		*Chrysomya albiceps* (wiedemann, 1819)	19	6.81
		*Lucilia illustris* (meigen, 1826)	4	1.43
		*Lucilia silvarum* (meigen, 1826)	1	< 1
		Calliphoridae sp.	6	2.15
	Drosophilidae	Drosophilidae sp.	2	< 1
	Fanniidae	*Fannia manicata* (meigen, 1826)	4	1.43
		*Fannia scalaris* (fabricius, 1794)	4	1.43
		*Fannia canicularis* (linnaeus, 1761)	2	< 1
		*Fannia* sp.	4	1.43
	Muscidae	*Musca domestica* (linnaeus, 1758)	10	3.58
		*Hydrotaea ignava* (harris, [1780])	8	2.87
		*Hydrotaea dentipes* (fabricius, 1805)	6	2.15
		*Hydrotaea aenescens* (wiedemann, 1830)	3	1.08
		*Muscina levida* (harris, [1780])	3	1.08
		*Hydrotaea capensis* (wiedemann, 1818)	2	< 1
		*Muscina prolapsa* (harris, [1780])	2	< 1
		*Hydrotaea similis* meade, 1887	1	< 1
		*Hydrotaea* sp.	8	2.87
		*Muscina* sp*.*	8	2.87
		Muscidae sp.	3	1.08
	Phoridae	*Megaselia scalaris* (loew, 1866)	13	4.66
		*Conicera tibialis* Schmitz, 1925	2	< 1
		*Megaselia abdita* Schmitz, 1959	1	< 1
		*Megaselia rufipes* (meigen, 1804)	1	< 1
		*Triphleba autumnalis* (becker, 1901)	1	< 1
		*Triphleba aequalis* (schmitz, 1919)	1	< 1
		Phoridae sp.	21	7.53
	Piophilidae	*Stearibia nigriceps* (meigen, 1826)	7	2.51
		Piophilidae sp.	4	1.43
	Sarcophagidae	*Sarcophaga argyrostoma* (robineau-desvoidy, 1830)	33	11.83
		Sarcophagidae sp.	12	4.3
	Scatopsidae	Scatopsidae sp.	1	< 1
	Sepsidae	*Nemopoda nitidula* (fallen, 1820)	2	< 1
	Sphaeroceridae	Sphaeroeridae sp.	1	< 1
	Stratiomyidae	Stratiomyidae sp.	1	< 1
	Syrphidae	*Syritta pipiens* (linnaeus, 1758)	2	< 1
		*Eristalis sp.*	2	< 1
	Trichoceridae	*Trichocera hiemalis* (de geer 1776)	1	< 1
Hymenoptera	Braconidae	Braconidae sp.	1	< 1
	Pteromalidae	*Nasonia vitripennis* (walker, 1836)	9	3.23

Blow flies (Diptera: Calliphoridae) were the most dominant insect family represented by ten taxa, eight of which were found frequently (minimum 19 cases). *Lucilia sericata* was by far the most abundant blow fly and colonized over 50% of all bodies, followed by *Calliphora vicina*, 39.78%, and *Lucilia ampullacea* 24.37%, respectively. For the beetles, *Dermestes lardarius* (found on 3.58% of the carcasses) was the most abundant species but was overall found less frequently than blow fly species. In addition to the blow flies, flesh flies (Diptera: Sarcophagidae) colonized human remains in 16.13% of all cases. In contrast to the high species diversity of the blow flies, the flesh flies were represented by only one species, *Sarcophaga argyrostoma*. Species of the family of scuttle flies (Diptera: Phoridae) were found in 14% of all cases, with *Megaselia scalaris* being most common. The last family of flies, which occurred frequently (19%), were the house flies (Diptera: Muscidae), with *Musca domestica* and *Hydrotaea ignava* being the most common species. However, most of those species, ~ 70%, were found in less than 10 cases.

### *Lucilia sericata*

*L. sericata* was by far the most abundant species and was found in over 50% of all cases in this study, making it to one of the most important species in forensic entomology in Germany as well as many other European countries [30–33]. It belongs to the green bottles (Luciliinae), so-called because of their brilliant metallic greenish coloration (Fig. 41a) and is small in size 5–10 mm [34]. The juvenile stages, maggots, are of cylindrical shape, whitish in colour (Fig. 41b), and develop (feed) in carrion (human, animal). It belongs to the first wave of colonizers of cadaver and can arrive minutes to hours after death, when the cadaver is still in the fresh stage (Fig. 41c). *L. sericata* is a high-summer, thermophilic, sun-loving species that is active from April until October with the main activity in the summer months from June to August [31]. It is overall considered to be a synanthrophic species closely associated with human habitations and urban areas [33]. In our study it was highly associated with indoor scenarios which underlines the synanthrophic nature of this species. *L. sericata* is not only one of the first colonizers of carrion, but can also cause severe cases of myiasis, i.e. the infestation of living people by fly larvae [35, 36]. In these cases, there is often neglect, both self-inflicted and by third parties. The development from egg to the adult fly takes 12.9 days at 25 °C [37] and stops below 10–15 °C [38, 39] and above 37 °C [40]. More information on the biology, i.e. developmental rates, seasonal activity and the importance in legal investigation, can be found in [30–33, 35, 38, 41–43].

**Fig. 4:**
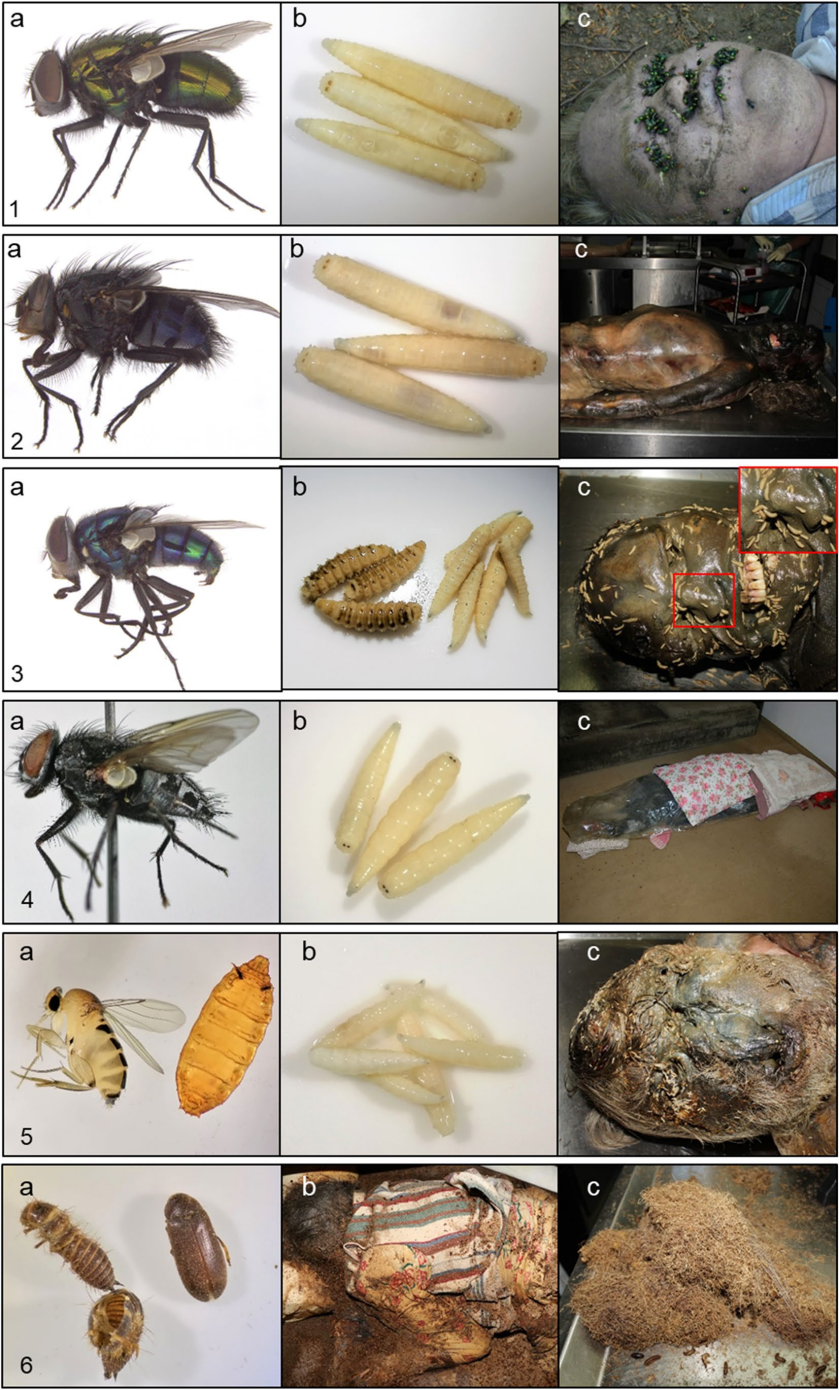
Pictures of the most relevant species in forensic entomological case work. **1** *Lucilia sericata*: adult fly; **b** 3rd instar larvae; **c** adult specimens colonizing natural orifices (eyes, mouth) of a fresh human body. **2** *Calliphora vicina*: **a** adult fly; **b** 3rd instar larvae; **c** mono-colonization on a human body of *C. vicina.* **3** *Chrysomya albiceps*: **a** adult fly; **b** 3rd instar larvae; **c** mono-colonization on a human body of *Ch. albiceps* with zoom on the characteristic of the larvae. **4** *Muscina prolapsa*: **a** adult fly, **b** 3rd instar larvae; **c** wrapped human body with colonization of *Muscina* species. **5** *Megaselia scalaris*: **a** adult fly and pupa; **b** 3rd instar larvae; **c** colonization of *M. scalaris* on a human body with pupae stick on the face. **6** Dermestidae: **a** adult beetle and exuviae; **b** body of a 93-year-old woman colonized by *Dermestes lardarius*; **c** excrements of larvae and adult larder beetles; fibrous horsehair-like, dark-brown material

### *Calliphora vicina*

*C. vicina* was the second most common species and colonized the bodies in almost 40% of all cases in this study, making it likewise to *L. sericata* one of the most relevant species in forensic entomology not only in Germany but also in the entire Holarctic region [21, 32, 34, 44]. It belongs to the bluebottles, which are large (5–12 mm [34]), blue metallic, bristly flies (Fig. 42a), associated with fresh cadavers*. C. vicina* is a cold-adapted species, which can develop even at 1 °C [45], and it is the only blow fly species in Germany being active, i.e. colonizing carcasses, all year round [31, 32]. It is particularly active in spring and autumn, i.e. during the colder periods of the year, and reduces its activity during the warmer periods in summer. Mono-colonization by *C. vicina* occurred in 24% of cases with colonization by this species (Fig. 42c), mainly in winter and spring. *C. vicina* is considered an optionally synanthrophic species since it inhabits both urban and natural ecosystems. However, in our study it was more often found in indoor scenarios (~ 70%). The development from egg to the adult fly takes 16.9 days at 25 °C [37] and stops below 1 °C [45] and above 30 °C. More information on the biology, i.e. developmental rates, seasonal activity and the importance in legal investigation, can be found in [31, 46, 47].

### *Chrysomya albiceps*

*Ch. albiceps* (Fig. 43a) was not one of the most abundant blow fly species (6.81% of all cases), but due to its unique morphological larval and puparial characteristics and biology as well as its seasonal activity, it can be important for PMI_min_ estimations in cases with long PMIs. This species is very common and abundant in Southern Europe, Afrotropical, Oriental and Neotropical regions [48] but was first found in Germany in 2001 [49]. Since this time, it started to expand slowly and was increasingly found on carcasses during the summer months. The morphological characteristics of the larvae, e.g. with prominent fleshy protrusions along their body, make it easy even for people not familiar with the morphology of flies to distinguish them from other blow fly species (Fig. 43b, c). Furthermore, the 2nd and 3rd instar larvae are predaceous, feeding on native blow fly larvae on a cadaver reducing their abundance drastically. The development from egg to the adult fly takes 12.5 days at 25 °C [37], and the development stops below 15 °C [48] and above 48.8 °C [50]. Currently, in Germany the activity of *Ch. albiceps* is limited to 3 months of the year, due to its high temperature requirement and can therefore serve as an indicator for colonization in July, August and September, even in cases of cadavers discovered after several months or years. It is expected that due to climate change, the species spectrum and its population dynamics may change rapidly in the future [51]. More information on the biology, i.e. developmental rates, seasonal activity and the importance in legal investigation, can be found in [30, 48, 49, 52, 53].

### *Muscina* species

In 4.66% of all cases, species of the genus *Muscina* colonized human carcasses. This number does not make it an abundant genus in forensic entomological casework, but due to its biology it can serve as an indicator for specific case scenarios such as burial, concealment and neglect, as well as a sign for the relocation of a body [54]. In all cases associated with species of the genus *Muscina* (*Muscina prolapsa* (Fig. 44a, b), *Muscina levida*), the location was specific, and the bodies were difficult for insects to access. There were cases where the bodies were buried up to 40 cm deep, hidden in bags or suitcases, covered with plastic sheets (Fig. 44c), clothes or garden waste, and one body was hidden in the trunk of a car. As a result, species of this genus can provide information on the crime scene scenario even if it has been subsequently modified, e.g. a body was stored in a trunk of a car, buried or concealed before it relocated to the place of finding. In addition, they can serve as an indicator in cases of neglect where people are not adequately cared for and often live under very poor hygienic conditions. The development, shown exemplarily for *Muscina prolapsa*, from egg to the adult fly takes 12.6 days at 25 °C [54] and stops below 6.7° C [54]. More information on the biology, i.e. developmental rates, seasonal activity and the importance in legal investigation, can be found in [54–59].

### Phoridae

In ~ 14% of all cases, species of the family Phoridae colonized the remains. Species of this family are minute to medium sized (0.75–8.00 mm), dull back, brown or yellowish flies of a humped back appearance [21] (Fig. 45a, b). On a carcass they can be recognized by their behaviour, of rather run fast to escape than fly. That is why they also called scuttle flies. The most common species on human carcasses in our study was *Megaselia scalaris*. Scuttle flies can enter narrow openings and colonize carcasses even when they are hard to access for other typical carrion breeders like blow flies. They can colonize buried [60] or concealed bodies that are wrapped in plastic or concealed in a trash cans or suitcases. Additionally, they are cold-adapted species active at temperatures at which blow flies are inactive [61]. They are associated with several stages of decomposition, i.e. ovipositing immediately after death or not before the late stages of decomposition, e.g. mummified bodies (Fig. 45c). In our study they were clearly associated with indoor crime scenes (~ 80%), as also reported by several other authors [61–63]. In our study it was commonly found in flats which have been in a disastrous state, i.e. very dirty. The development, shown exemplarily for *Megaselia scalaris,* from egg to the adult fly takes 12.6 days at 25 °C [64]. More information on the biology, i.e. developmental rates, seasonal activity and the importance in legal investigation, can be found in [33, 61–66].

### Dermestidae

In general, beetles play a rather minor role in forensic entomological case work in Germany due to their later appearance at a carcass and their main association to outdoor scenarios. However, especially beetles of the family larder beetles (Coleoptera: Dermestidae) can be important for the estimation of the PMI_min_ in cases of long PMIs and cases of mummified bodies (Fig. 4 6b). In our study, larder beetles were found in ~ 6% of all cases, all with PMIs from 3 months to several years. Larder beetles are small (0.5–1 cm) necrophagous beetles feeding on dried muscle tissue and skin [67]. The adult beetles have a characteristic oval shape, whereas the larvae are very prominent due long setae all along their body (Fig. 4 6a). Typical for a body colonized by larder beetles is fibrous horsehair-like, dark-brown material covering the entire carcass (Fig. 4 6b, c). These are the excrements of the adult beetles and larvae. Larder beetles colonize human cadavers in the dry, mummified stage in the absent of other carrion breeders like blow flies. In Germany, they are more active during the colder season and are more common indoors than outdoors. Under specific circumstances, e.g. hot and dry environment, rapid mummification of a body, absence of other necrophagous insects, they can also colonize a body in huge numbers in summer [68]. More information on the biology, i.e. developmental rates, seasonal activity and the importance in legal investigation, can be found in [23, 67–70].

### Seasonal oviposition activity

**Fig. 5:**
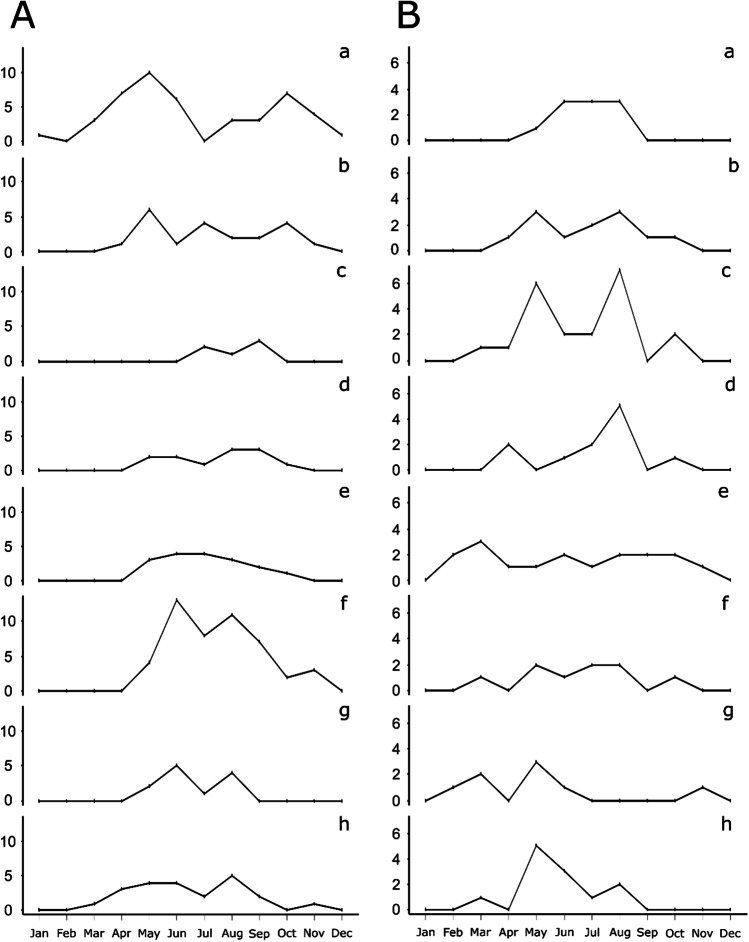
Seasonal oviposition activity on human bodies of **A** the most important blow flies (Diptera: Calliphoridae): **Aa** *C. vicina*, **Ab** *C. vomitoria*, **Ac** *Ch. albiceps*, **Ad** *L. ampullacea*, **Ae** *L. caesar*, **Af** *L. sericata*, **Ag** *P. regina*, **Ah** *P. terraenovae* and **B** the most important forensically relevant flies and beetles: **Ba** *S. argyrostoma*, **Bb** Fannia sp., **Bc** *Hydrotaea* sp., **Bd** *Muscina* sp., **Be** Phoridae sp., **Bf** Piophilidae sp., **Bg** Dermestidae sp., **Bh** Silphidae sp

The seasonal oviposition activity of the most important, i.e. most abundant species, was estimated based on the calculation of the PMI_min_ in the entomological reports (n = 139). The majority of blow fly species, e.g. *L. ampullacea* (Fig. 5ad), *L. caesar* (Fig. 5ae), *P. regina* (Fig. 5ag) and *L. sericata* (Fig. 5af), were exclusively active in the warm period of the year from May until September. *P. terraenovae* started its activity in February and was active until November (Fig. 5ah), thus showing a similar activity pattern as *C. vomitoria* (Fig. 5ab). *C. vicina* was the only species that was active all year round (Fig. 5aa), and unlike all other blow flies, it was more active in spring and autumn and decreased in activity during the summer months. A likewise unique seasonal oviposition activity showed *Ch. albiceps* (Fig. 5ac), characterized by colonizing human remains exclusively from July to September. Species of the families Fanniidae and Muscidae (*Hydrotaea*, *Muscina*) showed a similar seasonal activity as most blow fly species (Fig. 5bb–d). The species were active in the warm period of the year from April until October. The only flesh fly species, *S. argyrostoma*, was clearly associated with the summer months and colonized the bodies from May until August (Fig. 5ba). Species of Phoridae and Piophilidae did not show clear seasonal preferences and colonized the bodies throughout the year (Fig. 5be–f). For the most abundant beetle families, Dermestidae and Silphidae, two different seasonal patterns were observed. Species of Dermestidae were more abundant (active) in the beginning of a year until May and decreased in activity throughout the summer months (Fig. 5bg), whereas species of Silphidae were almost exclusively active in the warmer months of the year, i.e. March to August with a peak in May (Fig. 5bh).

### Habitat preference

**Fig. 6:**
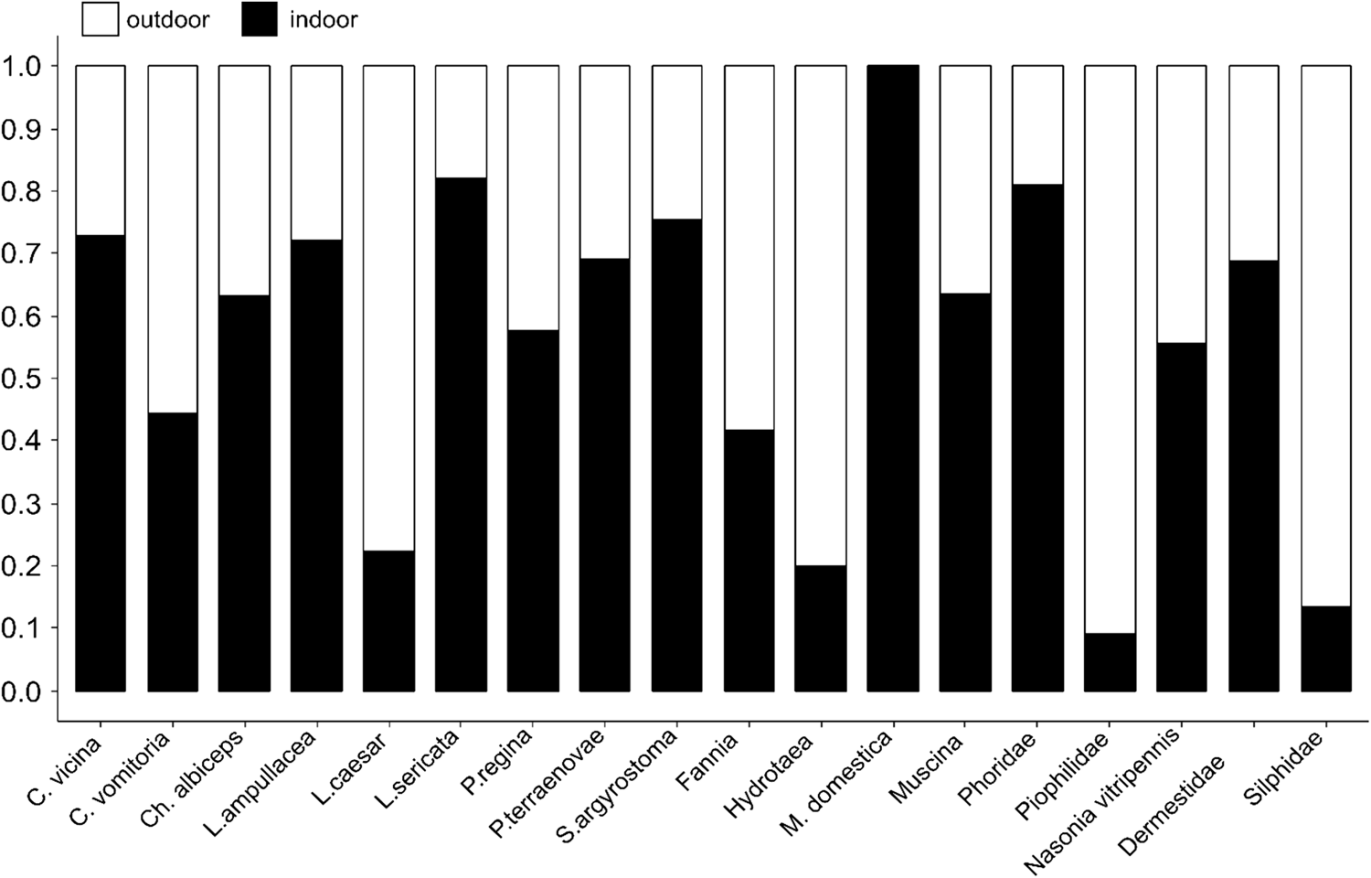
Percentage distribution of indoor (black) and outdoor (white) cases for the most important and abundant species

Some of the most important and abundant species showed a habitat (indoor/outdoor) preference (Fig. 6). The flesh fly *S. argyrostoma* and most blow fly species were strongly (*S. argyrostoma*, *L. sericata*, *C. vicina*) or slightly (*L. ampullacea*, *Ch. albiceps*) associated with indoor scenes. *C. vomitoria* and *Fannia* spp. tend to appear more frequently on bodies located outdoors, while *L. caesar*, *Hydrotaea* and species of the family Piophilidae showed a clear preference for outdoor crime scenes. Further species with an association to indoor crime scenes were *Muscina* spp., species of Phoridae and dermestid beetles. One species, *Musca domestica,* was exclusively found on bodies located indoors, whereas species of the family Silphidae were almost exclusively found outdoors.

By using the information on biology, activity and distribution of the relevant species and families, we were able to establish a PMI_min_ in many of the requested cases. The following case descriptions illustrate this clearly for the 5 target categories indicated in Table 2.

## Example cases for the different PMI groups

### PMI 1–7 d

On 12 June, the severely decomposed body of a woman, who had been missing since 8 days, was discovered in a heavily overgrown green area on a former quarry. The entomofauna on the body was dominated by the blow fly *L. caesar*. All larval specimens were still in their feeding stage and had not yet left the body. The reconstructed mean temperature of the 8 days prior to the discovery of the body (24.5 °C) was used in the entomological assessment. By applying data by Smith [21] and Richards & Hall (unpublished, personal communication), it was estimated that the body of the woman was first colonized by *L. caesar* between 6 and 7 June, resulting in a PMI_min_ of 5–6 days. Based on the police investigation, the actual time of death was in the night of 5 June–6 June, i.e. a PMI of 7 days, which is also supported by the entomological report.

### PMI > 1–3 w

On 24 July, an advanced skeletonized body was discovered in an apartment. The tenant of the apartment was last seen alive on 7 July. Mainly larvae, pupae and empty puparia of flies were sampled during autopsy. All developmental stages of flies were identified as the blow fly *P. terraenovae*. No temperature measurements were available from the apartment, but following the data from the national meteorological service (NMS) high activity by blow flies was to be expected especially in the days immediately after 7 July. Due to the lack of sound measurements on site, two different temperatures (20 °C and 25 °C) were used as the basis for the development of blow flies, which were intended to reflect the range of possible room temperatures in an apartment. According to Grassberger [71], *P. terraenovae* needs about 20 days at ~ 20 °C and about 15 days at 25 °C to complete the development from egg to the adult fly, which indicates a colonization between 4 July (assuming 20 °C) and 9 July (assuming 25 °C). Together with the information on when the man was last seen alive, this narrowed down the period of death to the 7 July–9 July, giving an estimated PMI_min_ of 15 days. This was later confirmed by the police investigation.

### PMI > 3 w–3 m

On 22 December, a body was found in an abandoned garden spot in an advanced state of decomposition, covered with various blankets. Initial police investigation revealed that the deceased was a ~ 50-year-old man who lived in the house belonging to that garden area. The man was last seen alive on 20 October. Autopsy revealed at least two blunt violent impacts against the right side of the skull. There was a massive infestation of fly larvae on the body, and various fly pupae were also found on the corpse itself and its covering. No temperature measurements were taken at the discovery site for a direct comparison between the location of the body and the location of the nearest weather station. Hence, temperature recordings from a NMS weather station were used. Since 24 October, the temperature was not above 13 °C with an average of 5.1 °C, rarely reaching sub-zero temperatures until the day of discovery on 22 December. Numerous maggots (all in the third/last larval stage) and pupae were sampled; the latter showed already advanced colouration and first characteristics of adult flies such as body segmentation, developed legs and bristles. The samples were identified as the blow flies *C. vicina* and *C. vomitoria*. According to Marchenko [38], and assuming that the metamorphosis of the flies was very advanced, colonization of the body by the two fly species took place between 20 and 29 October, i.e. closely after the disappearance of the victim, giving an estimated PMI_min_ of about 2 months.

### PMI > 3 m–6 m

On 14 November, two partially skeletonised male corpses were found in a small stand of trees in a park area. The bodies were laying in sleeping bags and were dressed in T-shirts and pants. The skulls were fractured in the face and forehead area, so that a homicide had to be assumed. There were few living fly larvae but numerous empty fly puparia in the sleeping bags and soil beneath the corpses. Apart from a few specimens of the blow fly species *Lucilia* sp. and *Calliphora* sp., almost all empty puparia belonged to the invasive blow fly *Ch. albiceps*. More fly species were found in their larval and pupal stages in the soil, namely *H. ignava*, *F. scalaris*, *F. manicata* and *S. nigriceps*. There were also fragments of puparia of flesh flies and fruit flies. *Ch. albiceps* needs special temperature conditions with regard to oviposition and larval development, and it was expected that suitable temperature conditions for oviposition and development exist from mid-May until the end of August in Germany. However, the presence of different later colonizing species in their larval and pupal stages at the time of the discovery in mid-November excludes the possibility of a PMI of more than 3–4 months. Altogether a first insect colonization between middle of July and middle of August and a PMI_min_ of about 3 months seems most likely. Later, the police investigation revealed that the murder of the two men probably took place in the middle of August.

### PMI > 6 m

On 1st October, the body of a female was found in the bed of her apartment. There were no signs of violence and the autopsy could not clarify the cause of death. The body was dressed with an adult diaper, was in a cachectic general condition, and in a state of advanced mummification, weighing only 13 kg. A sore was provisionally treated with plaster bandages. On the basis of the statements of neighbours and the newspapers found in the apartment, the responsible public prosecutor’s office assumes that the female’s partner, who disappeared, stayed in the apartment at irregular intervals for several months before the day of the discovery. Hundreds of empty puparia were sampled, all belonging to the Muscidae and representing the genera *Muscina* sp. and *Hydrotaea* sp. No blow flies or other typical first colonizers were found. In some of the empty puparia, there were clear traces of silk, proofing the presence of moth larvae using the empty puparia as a shelter for producing a cocoon.

Overall, the entomological findings indicated a death several months ago. Especially the lack of blow flies, typically active between March and October, suggested that death occurred already at least the winter before, therefore indicating a PMI_min_ of more than 6 months.

## Conclusion

Forensic entomology has a long history of research and application and is one of the most important tools in court proceedings when it comes to PMI estimations (Gelderman et al. submitted), not to mention its enormous importance in homicide investigations, especially during the first days. Knowing the (minimum) time since death as soon as possible helps to direct the next steps of the investigation in the right direction.

Our analysis of almost 1000 cases showed: When insects are present, they are one of the most powerful tools for determining a PMI_min_ and narrowing down a possible time since death, with minimal sampling effort. Hence, one would expect that forensic entomology has accordingly undergone a heroic triumph in the forensic sciences — but this is not the case. This is illustrated by the literature and case reports on PMI estimates via, e.g. total body scores, in which entomological evidence is noted and described but not used and analysed as relevant evidence [72–78], or by the statement of a retired director of an Institute of legal medicine, who mentions (personal communication) that entomological analyses were not necessary in the decades of his professional activity. Such kind of ignorance is a human stigma from which every profession can suffer, i.e. not a specific problem of forensic entomology. Nevertheless, we found three main problems of relevance, inherent to the system, which prevent breakthrough and necessary acceptance of forensic entomology which also been touched upon by other studies focusing on best practice and sampling [79–83]. Firstly, all investigators, forensic biologists, crime scene technicians, forensic pathologists, etc. should know forensic entomology and be aware of the potential power of insects in an investigation. Not knowing about the use of insects in a homicide investigation is unacceptable or even malpractice. Hence, relevant institutions (police academies, universities, etc.) should implement forensic entomology in their curriculum.

Secondly, estimating the time since death by the means of insects is a multidisciplinary approach when it comes to sampling and storing the evidence at the scene of death or during the autopsy — many different professions might be responsible for securing the evidence at different points in time during the recovery and examination of the corpse. Such different responsibilities can be an obstacle. Just thinking that “all this sampling will be done later during the autopsy by the forensic pathologist” (as all insects seems to be on the dead body) could lead to a wrong or incomplete sampling. Here, the prompt involvement of an entomologist, even if only in an advisory role via telephone or social media, can substantially improve the securing of evidence especially at the scene of death.

Thirdly, the evaluation of entomological evidence requires expert knowledge. Forensic entomology is an independent discipline; this means that a forensic entomologist should analyze the evidence, write the report and explain the results eventually also in court during the trial. The success of an entomological report depends largely on the acceptance of this fact. Nevertheless, the quality of the report is strongly influenced by an interdisciplinary cooperation of forensic pathologists, investigators and entomologists.
